# Comprehensive Exploration of M2 Macrophages and Its Related Genes for Predicting Clinical Outcomes and Drug Sensitivity in Lung Squamous Cell Carcinoma

**DOI:** 10.1155/2022/1163924

**Published:** 2022-09-14

**Authors:** Yansong Han, Yuexia Li

**Affiliations:** Department of Pharmacy, The Second Affiliated Hospital Haybin Medical University, Haybin, China

## Abstract

**Background:**

M2 macrophages play an important role in cancers. However, the role of M2 macrophages has not been clarified in lung squamous cell carcinoma.

**Methods:**

All the open-accessed data were downloaded from The Cancer Genome Atlas database. All the analysis was performed in the R software. The CIBERSORT algorithm was utilized to quantify the immune cell infiltration in the tumor microenvironment. LASSO regression and multivariate Cox regression analysis were carried out for the creation of the prognostic model. Pathway enrichment analysis was performed using the single sample Gene Set Enrichment Analysis (ssGSEA) and clueGO algorithm.

**Results:**

In our study, we comprehensively explored the role of M2 macrophages and its related genes in LUSC patients. We found that the patients with high M2 macrophage infiltration tend to have a worse prognosis. Also, some oncogenetic pathways were activated in the patients with high M2 macrophage infiltration. Further, a prognosis model based on six M2 macrophage-related genes was established, including TRIM58, VIPR2, CTNNA3, KIAA0408, CLEC4G, and MATN4, which showed a good prognosis prediction efficiency in both training and validation cohort. Pathway enrichment analysis showed that the pathway of allograft rejection, bile acid metabolism, coagulation, inflammatory response, IL6/JAK/STAT3 signaling, hedgehog signaling, peroxisome, and myogenesis were significantly activated in the high-risk patients. Based on the results of an investigation of immune infiltration, risk score was found to have a positive correlation with M2 macrophages and resting CD4+ memory T cells, but a negative correlation with follicular helper T cells, M1 macrophages, and Tregs. In addition, we discovered that patients in high-risk groups may respond better to immunotherapy than individuals in lower-risk groups. However, low-risk patients might be more sensitive to cisplatin.

**Conclusions:**

Our model is a powerful tool to predict LUSC patient prognosis and could indicate the sensitivity of immunotherapy and chemotherapy.

## 1. Introduction

Lung cancer is a common malignant tumor globally and is approximately responsible for 2 million new cases and 1.5 million cancer-related deaths each year [1]. The incidence of lung cancer is related to several pathogenesis factors, including environmental exposure, smoking, lifestyle, and genetic predisposition [2]. Non-small-cell lung cancer (NSCLC) is the most frequently pathological subtype of lung cancer, mainly consisting of lung adenocarcinoma (LUAD) and lung squamous cell carcinoma (LUSC) [3, 4]. For the moment, surgery resection is still the best option for early-stage lung cancer. However, the overall survival rate for advanced lung cancer remains unsatisfactory, despite immunotherapy and targeted therapy providing some therapeutic benefits [5, 6]. Consequently, in order to diagnose and treat LUAD, it is essential to discover new molecular markers that are both effective and innovative.

Tumor cells are affected by various factors in the tumor microenvironment, rather than being isolated individuals [7]. Macrophages are a member of the tumor microenvironment and have been reported to affect tumor progression through intercellular interactions, secretion of cytokines, and other effects [8]. Generally speaking, macrophages are classified into three groups: M0, M1, and M2 types, of which M1 and M2 are different from M0 macrophages [8]. M2 macrophages have been widely reported to be involved in tumor development [9]. For example, Lan and colleagues found that the M2 macrophage-derived exosomes miR-21-5p and miR-155-5p could significantly promote cancer cell invasion and are potential therapeutic targets for colon cancer [10]. Weng and colleagues found that the polarization process of M2 macrophages could be induced by the MCT-1/miR-34a/IL-6/IL-6R axis, therefore facilitating breast cancer progression [11]. Wang and colleagues revealed that tumor-derived exosome miR-301a was induced by hypoxia and can mediate M2 Macrophage polarization through PTEN/PI3K*γ* pathway to enhance pancreatic cancer metastasis [12]. Therefore, it is meaningful to explore the underlying role of M2 macrophages and its related molecules in lung cancer.

In our study, we aimed to develop a novel prognostic model for LUSC patients. We comprehensively explored the role of M2 macrophages and its related genes in LUSC patients. Meanwhile, a prognosis model based on six M2 macrophage-related genes was established, including TRIM58, VIPR2, CTNNA3, KIAA0408, CLEC4G, and MATN4, which showed a good prognosis prediction efficiency. In order to investigate the underlying clinical and biological differences that exist between patients with a high risk and patients with a low risk, clinical correlation, route enrichment, and immune infiltration studies were carried out. In addition, we discovered that patients in high-risk groups may respond better to immunotherapy than individuals in lower-risk groups. However, low-risk patients might be more sensitive to cisplatin.

## 2. Materials and Methods

### 2.1. Data Acquisition

The open-accessed data of LUSC patients were obtained from TCGA datasets. Detailed, the transcriptional profile data were in “STAR-Counts” form and were integrated with R code. Clinical information of each patient were downloaded in “bcr-xml” form. All data were preprocessed before data analysis.

### 2.2. Immune Cell Infiltration and Identification of M2 Macrophage-Related Genes

The CIBERSORT algorithm was utilized to quantify the immune cell infiltration in the tumor microenvironment [13]. The CIBERSORT port is a general calculation method, which is used to quantify the immune cell fraction from the tissue gene expression profile, and can accurately estimate the immune component of tumor biopsy. Limma package was used to perform differentially expressed genes (DEGs) analysis between patients high and low M2 macrophage infiltration with the threshold of |logFC| > 1 and *P* < 0.05. The DEGs above were defined as M2 macrophage-related genes.

### 2.3. Pathway Enrichment and Genomic Analysis

Pathway enrichment analysis was performed using the single sample Gene Set Enrichment Analysis (ssGSEA) and clueGO algorithm to explore the underlying biological differences between two specific groups [14]. The reference pathway set was Hallmark. The TMB, MSI, and tumor stemness scores were obtained from TCGA.

### 2.4. Prognosis Model Construction

In the first step of the process, patients were randomly assigned to either the training or validation cohort. A univariate Cox regression analysis was carried out in order to locate the genes associated with the prognosis. After that, LASSO regression and multivariate Cox regression analysis were carried out for the creation of the prognostic model with the formula of “risk score = gene A∗Coef A + gene B∗Coef B + ⋯+gene N∗Coef N” [15, 16]. For the purpose of model evaluation, Kaplan-Meier survival curves and Receiver Operating Characteristic (ROC) curves were utilized.

### 2.5. Immunotherapy and Drug Sensitivity Assays

The Tumor Immune Dysfunction and Exclusion (TIDE) algorithm was used to measure the efficacy of immunotherapy for patients with LUSC [17]. The Genomics of Drug Sensitivity in Cancer database served as the basis for the drug sensitivity analysis that was carried out [18].

### 2.6. Statistical Analysis

All the statistical analyses were performed using the R software v4.0.0. For the data that had a normal distribution, a Student *T*-test was carried out. When analyzing data with a nonnormal distribution, the Mann–Whitney *U* test was utilized. *P* < 0.05 was considered statistically significant.

## 3. Results

### 3.1. Quantification of M2 Macrophages in LUSC

**Figure 1:**
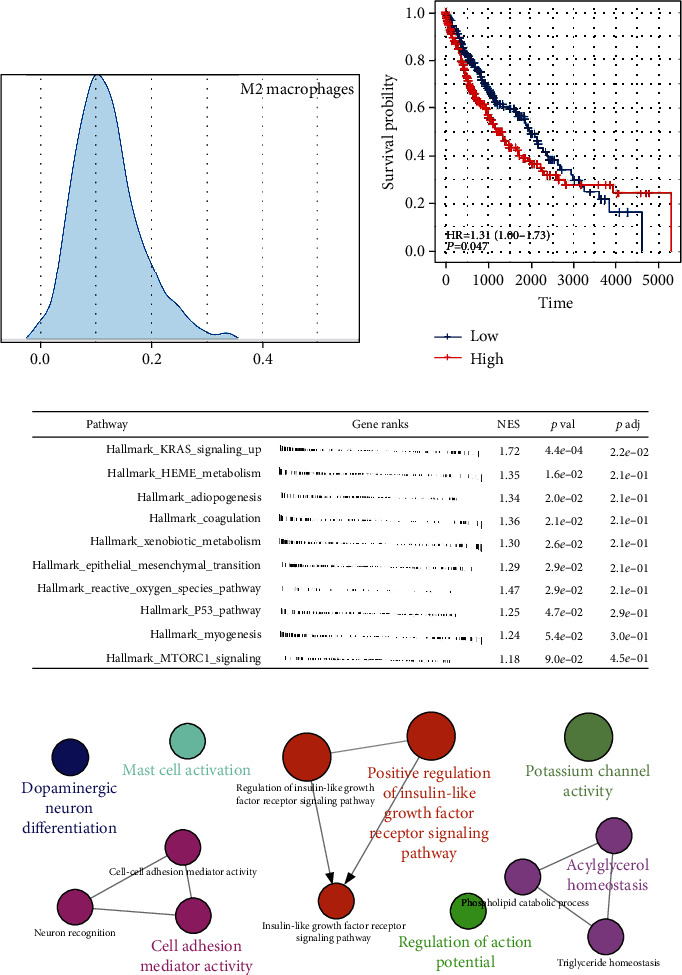
M2 macrophages in LUSC. (a) The infiltration level of M2 macrophages was quantified using the CIBERSORT algorithm; (b) the patients with higher M2 macrophage infiltration tend to have a worse prognosis; (c) pathway enrichment analysis M2 macrophages; (d) ClueGO analysis.

In the patients with LUSC, the infiltration level of M2 macrophages was measured using the CIBERSORT method (Figure 1(a)). KM survival curves showed that the patients with high M2 macrophage infiltration tend to have a worse prognosis (Figure 1(b)). Limma package was used to perform differentially expressed gene (DEG) analysis between patients high and low M2 macrophage infiltration with the threshold of |logFC| > 1 and *P* < 0.05. A total of 81 downregulated genes and 38 upregulated genes were defined as M2 macrophage-related genes (Figure S1). Pathway enrichment analysis showed that in the patients with high M2 macrophage infiltration, the pathway of KRAS signaling, HEME metabolism, adipogenesis, coagulation, xenobiotic metabolism, and epithelial-mesenchymal transition (EMT) were significantly enriched (Figure 1(c)). ClueGO analysis showed that the DEGs between high and low macrophages were mainly involved in dopaminergic neuron differentiation, mast cell activation, cell adhesion mediator activity, positive regulation of insulin-like growth factor receptor signaling pathway, regulation of action potential, potassium channel activity, and acylglycerol homeostasis (Figure 1(d)).

### 3.2. Prognosis Model Construction

**Figure 2:**
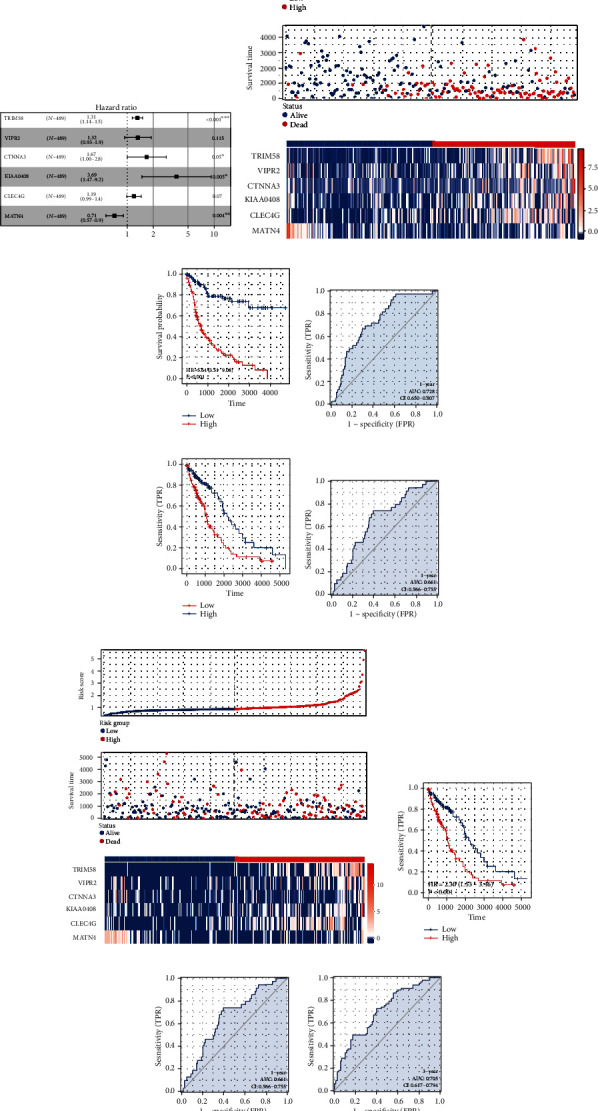
Prognosis model construction. (a, b) LASSO regression algorithm; (c) multivariate Cox regression analysis; (d) overview of the prognosis model in the training cohort; (e) KM survival curve of the model in the training cohort; (f–h) ROC curve was used for model evaluation in the training cohort; (i) overview of the prognosis model in the validation cohort; (j) KM survival curve of the model in the validation cohort; (k–m) OC curve was used for model evaluation in the validation cohort.

**Figure 3:**
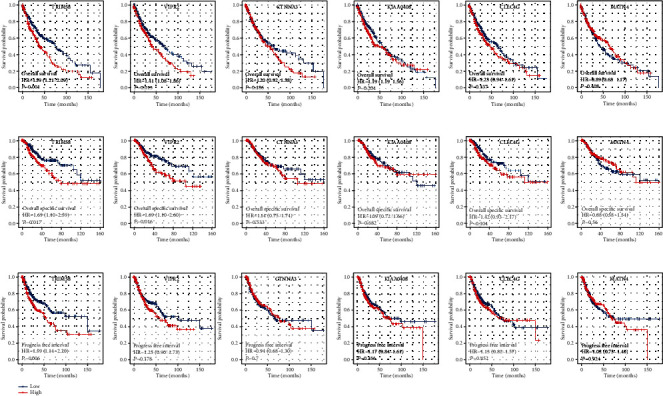
Prognosis effect of model genes. (a) The OS difference in patients with high and low model gene expression; (b) the DSS difference in patients with high and low model gene expression; (c) the PFS difference in patients with high and low model gene expression.

A univariate Cox regression analysis was performed as the first step of the method in order to discover the genes that were connected with the prognosis. After that, LASSO regression was carried out in order to reduce the dimensionality of the data (Figures 2(a) and 2(b)). Multivariate Cox regression analysis finally identified six genes for model construction, including TRIM58, VIPR2, CTNNA3, KIAA0408, CLEC4G, and MATN4 (Figure 2(c)). Within the training group, a larger percentage of fatalities was seen in patients who were considered to be at high risk (Figure 2(d)). KM survival curve showed that the patients in the high-risk group had a worse overall survival (OS) (Figure 2(e)). ROC curves indicated a good prediction of patients 1-, 3-, and 5-year OS (Figures 2(f)–2(h), 1‐year AUC = 0.728, 3‐year AUC = 0.75, and 5‐year AUC = 0.81). The same trend was also found in the validation cohort (Figures 2(i)–2(m), 1‐year AUC = 0.661, 3‐year AUC = 0.705, and 5‐year AUC = 0.746). Next, we explored the prognosis value of six model genes. The result showed the genes TRIM58, VIPR2, CTNNA3, KIAA0406, and CLEC4G might be associated with poor OS and disease-free survival (DSS), while MATN4 was associated with better prognosis (Figures 3(a) and 3(b)). Meanwhile, the patient with higher TRIM58 expression might have a shorter progression-free survival (Figure 3(c)).

### 3.3. Clinical Correlation Analysis

**Figure 4:**
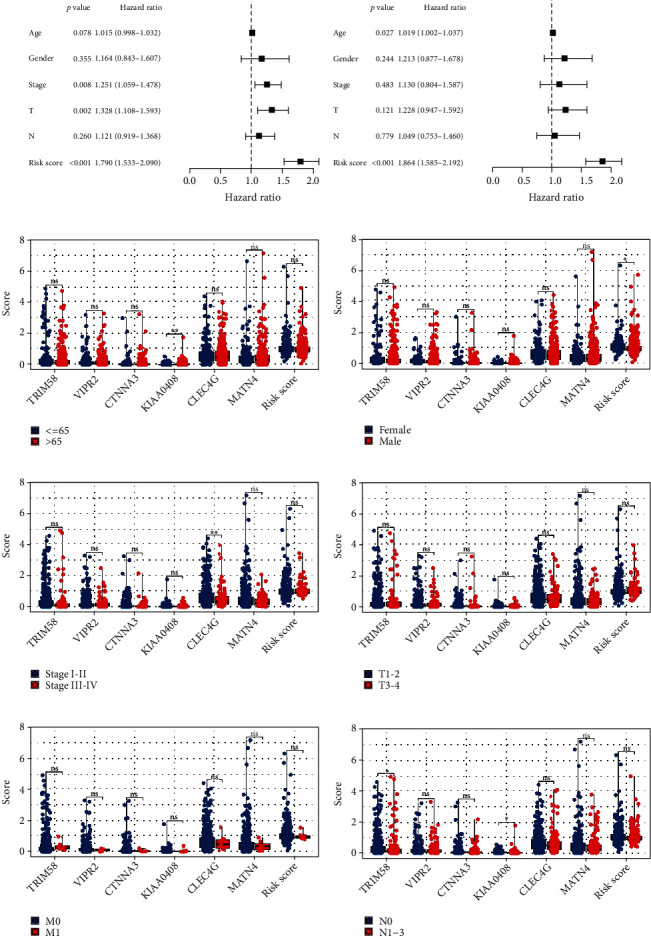
Clinical correlation analysis. (a, b) Both univariate and multivariate analyses were performed on the risk score and clinical characteristics; (c–h) clinical correlation of model genes and risk score.

Both univariate and multivariate analyses demonstrated that our model is a risk factor that is not reliant on any other clinical characteristics, including age, gender, T classification, N classification, and clinical stage (Figure 4(a), univariate analysis, HR = 1.79, *P* < 0.001; Figure 4(b), multivariate analysis, HR = 1.864, *P* < 0.001). Clinical correlation analysis indicated no significant difference of model genes and risk score in patients of different ages (Figure 4(c)). Interestingly, we found that the female patients might have a higher risk score than male patients (Figure 4(d)). Also, we observed a lower CLEC4G level in patients with a worse clinical stage (Figure 4(e)). No significant difference of model genes and risk score was observed in patients of different TNM classifications (Figures 4(f)–4(h)).

### 3.4. Pathway Enrichment, Immune Infiltration, and Genomic Instability Analysis

**Figure 5:**
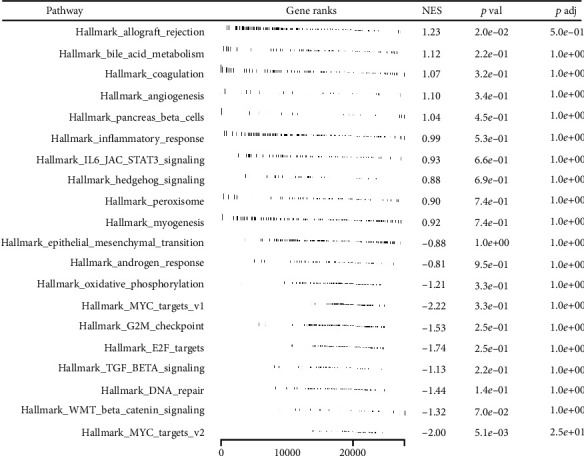
Pathway enrichment analysis.

**Figure 6:**
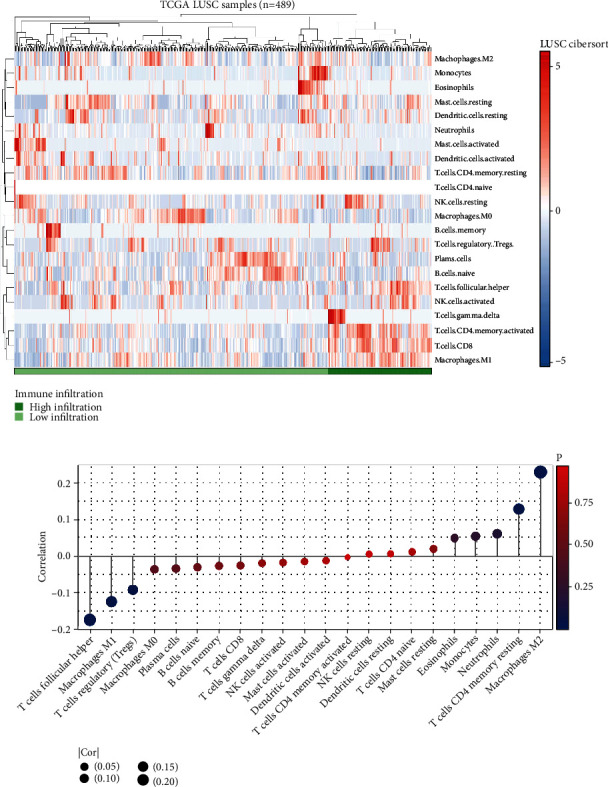
Immune infiltration analysis. (a) In order to quantify the immunological microenvironment, the CIBERSORT algorithm was utilized; (b) the correlation of risk score and quantified immune cells.

**Figure 7:**
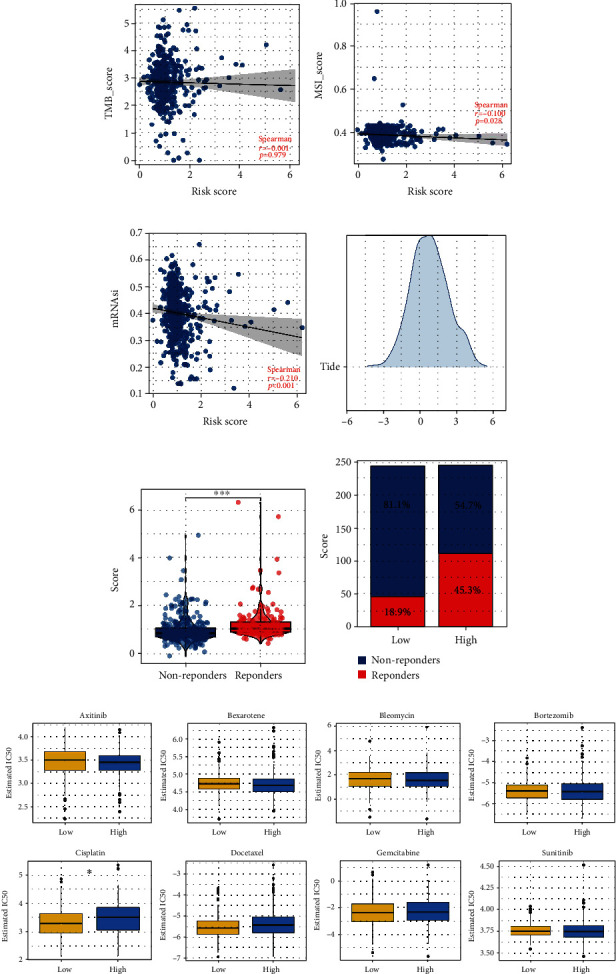
Immunotherapy and drug sensitivity analysis. (a) The correlation between risk score and TMB; (b) the correlation between risk score and MSI; (c) the correlation between risk score and mRNAsi; (d) TIDE algorithm was used to quantify the immunotherapy response; (e) the TIDE score in low- and high-risk patients; (f) the proportion of immunotherapy responders and non-responders in low- and high-risk patients; (g) drug sensitivity differences between low- and high-risk patients.

Moreover, we made an effort to determine the potential variations in biological pathways that exist between patients who have a high risk and those who have a low risk. The result showed that the pathway of allograft rejection, bile acid metabolism, coagulation, inflammatory response, IL6/JAK/STAT3 signaling, hedgehog signaling, peroxisome, and myogenesis were significantly activated in the high-risk patients (Figure 5). The CIBERSORT algorithm was used to quantify the immune microenvironment of LUSC patients (Figure 6(a)). Based on the results of an investigation of immune infiltration, risk score was found to have a positive correlation with M2 macrophages and resting CD4+ memory T cells, but a negative correlation with follicular helper T cells, M1 macrophages, and Tregs (Figure 6(b)). Genomic instability analysis showed that risk score had no remarkable correlation with TMB and MSI (Figures 7(a) and 7(b)). However, we found a negative correlation between risk score and mRNAsi (Figure 7(c)).

### 3.5. Immunotherapy and Drug Sensitivity Analysis

Both immunotherapy and chemotherapy were considered to be the most essential treatment options for lung cancer. Next, we investigated the underlying variations in immunotherapy and chemotherapy sensitivity between patients with a high chance of developing the disease and those with a low risk. The TIDE algorithm was used to quantify the immunotherapy response rate of LUSC patients (Figure 7(d)). The result showed that the immunotherapy responders might have a higher risk score (Figure 7(e)). Also, the patients in the high-risk group might have a higher proportion of immunotherapy responders (Figure 7(f)). The results of the drug sensitivity test suggested that people in the low-risk group would be more susceptible to the effects of cisplatin (Figure 7(g)). However, no significant difference was found in axitinib, bexarotene, bleomycin, bortezomib, docetaxel, gemcitabine, and sunitinib (Figure 7(g)).

## 4. Discussion

Lung cancer, the leading cause of cancer death throughout the world, claims the lives of more than 350 people every day [19]. During the last decade, although there was a steep decline in lung cancer incidence for advanced cases based on the changes in cancer screening and treatment, only 15% of patients with NSCLC can live beyond five years [20]. LUSC, the most common pathological subtype of NSCLC, known for its high tumor heterogeneity, showed a significant therapeutic difference in immunotherapy response rate [21]. A key objective of our research is to identify prognostic and therapeutic targets for LUSC.

In this study, we comprehensively explored the role of M2 macrophages and its related genes in LUSC patients. Meanwhile, a prognosis model based on six M2 macrophage-related genes was established, including TRIM58, VIPR2, CTNNA3, KIAA0408, CLEC4G, and MATN4, which showed a good prognosis prediction efficiency in both training and validation cohort. In order to investigate the underlying clinical and biological differences that exist between patients with a high risk and patients with a low risk, clinical correlation, route enrichment, and immune infiltration studies were carried out. In addition, we discovered that patients in high-risk groups may respond better to immunotherapy than individuals in lower-risk groups. However, low-risk patients might be more sensitive to cisplatin.

M2 macrophage is an essential part of the tumor microenvironment. In our study, we identified six model genes TRIM58, VIPR2, CTNNA3, KIAA0408, CLEC4G, and MATN4 that were associated with patients' prognosis and M2 macrophage infiltration. In lung cancer, Chen et al. found that TRIM58 was a prognostic biomarker that could remodel the tumor microenvironment of lung cancer [22]. Based on the whole-exome sequencing, Liu et al. indicated that CTNNA3 has the potential to be a promising druggable target in LUSC therapy [23]. However, there have been few studies examining the role of other genes playing in lung cancer, as well as their underlying association with M2 macrophage infiltration. The result of our study could provide a novel insight into the research direction of model genes.

The result in the present study showed that the pathway of allograft rejection, bile acid metabolism, coagulation, inflammatory response, IL6/JAK/STAT3 signaling, hedgehog signaling, peroxisome, and myogenesis were significantly activated in the high-risk patients. Through the large-scale metabonomic analysis of cancer tissue and plasma, Nie et al. found that bile acid metabolism was correlated with poor clinical features, which might be an underlying therapeutic target in lung cancer [24]. Meanwhile, Tantawy et al. found that the imbalance of IL6/JAK/STAT3 pathway and its related downstream pathways is the main reason for the progression of NSCLC [25]. The abnormal activation of the hedgehog pathway is responsible for causing and progressing several types of cancer [26]. In lung cancer, the hedgehog pathway was considered associated with lung cancer development [27]. Meanwhile, risk score was found positively correlated with M2 macrophage infiltration. In lung cancer, high level of M2 macrophages was associated with more progressive biological behavior. From our result, it is possible that the aberrant activation of the pathways mentioned above, along with the link with M2 macrophages, is to blame for the dismal prognosis of patients who fall into the high-risk group.

Nowadays, immunotherapy and chemotherapy were important therapy options for lung cancer. According to the findings of our study, patients who were in the high-risk group would respond better to immunotherapy, whereas those who were in the low-risk group might respond better to cisplatin. Therefore, aside from predicting the prognosis of lung cancer patients, our model also provides some therapeutic guidance. In the clinical setting, the application of our model could indicate the therapy option of LUSC patients.

It is important to be aware of some restrictions. Firstly, the majority of people who participated in our research were from Western countries. The underlying race bias might hamper the credibility of the application of our model to other races. Secondly, the M classification information of a considerable part of the population is unknown. If all clinical information is complete, our data will be richer and more reliable. Moreover, further experimental research is required to elucidate the protein expression levels of the prognostic genes as well as their molecular mechanisms in the pathogenesis and progression of LUSC.

## 5. Conclusion

Our study identified a novel signature that reliably predict overall survival in pancreatic cancer. The findings may be beneficial to therapeutic customization and medical decision-making.

## Data Availability

The data used in this research are available from the corresponding author upon reasonable request.

## Supplementary Materials

Supplementary Materials Figure S1: identification of M2 macrophages-related genes.
