# Supplementary figures and images for: Comprehensive Exploration of M2 Macrophages and Its Related Genes for Predicting Clinical Outcomes and Drug Sensitivity in Lung Squamous Cell Carcinoma
Source: J Oncol. 2022 Sep 14;2022:1163924. doi: 10.1155/2022/1163924 (PMC9492411; doi:10.1155/2022/1163924)

low vs high M2 macro

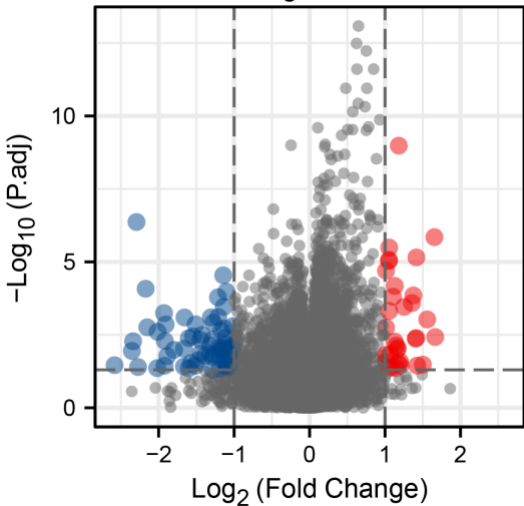

Supplement: Supplementary Materials — Figure S1: identification of M2 macrophages-related genes. [file 1163924.f1.pdf]
